# A Cooperative Traffic Control of Vehicle–Intersection (CTCVI) for the Reduction of Traffic Delays and Fuel Consumption

**DOI:** 10.3390/s16122175

**Published:** 2016-12-17

**Authors:** Jinjian Li, Mahjoub Dridi, Abdellah El-Moudni

**Affiliations:** Laboratoire Systèmes et Transports, Université de Technologie de Belfort-Montbéliard, Belfort 90000, France; mahjoub.dridi@utbm.fr (M.D.); abdellah.el-moudni@utbm.fr (A.E.-M.)

**Keywords:** dynamic programming, V2I, traffic delays, fuel consumption, speed profile

## Abstract

The problem of reducing traffic delays and decreasing fuel consumption simultaneously in a network of intersections without traffic lights is solved by a cooperative traffic control algorithm, where the cooperation is executed based on the connection of Vehicle-to-Infrastructure (V2I). This resolution of the problem contains two main steps. The first step concerns the itinerary of which intersections are chosen by vehicles to arrive at their destination from their starting point. Based on the principle of minimal travel distance, each vehicle chooses its itinerary dynamically based on the traffic loads in the adjacent intersections. The second step is related to the following proposed cooperative procedures to allow vehicles to pass through each intersection rapidly and economically: on one hand, according to the real-time information sent by vehicles via V2I in the edge of the communication zone, each intersection applies Dynamic Programming (DP) to cooperatively optimize the vehicle passing sequence with minimal traffic delays so that the vehicles may rapidly pass the intersection under the relevant safety constraints; on the other hand, after receiving this sequence, each vehicle finds the optimal speed profiles with the minimal fuel consumption by an exhaustive search. The simulation results reveal that the proposed algorithm can significantly reduce both travel delays and fuel consumption compared with other papers under different traffic volumes.

## 1. Introduction

Massive traffic delays and high fuel consumption are two serious traffic problems in our daily life, causing people to waste a lot of time and fuel every year. For example, Americans lost 4.8 billion h and 3.9 billion liters of gasoline in 2009 [1]. For cities where it is difficult to further construct and expand the roads, one of the most effective solutions is the efficient exploitation of the existing road resources by innovative traffic control. In conventional traffic control, the vehicles are guided by traffic lights, and do not catch the signal until near the intersection. As a result, vehicles cannot effectively adjust their speed profiles in advance to avoid stopping and idling before the intersection. Then, the traffic efficiency in the intersection is limited. There are various types of traffic control strategies in intersections with traffic lights, such as Fixed Time control (FT), Traffic Adaptive control, and Actuated Signals control. In FT control, the parameters of the control system (such as the phase sequence and the period for each phase) are based on the historical average traffic flow during the same period. As a result, FT control cannot catch the change of traffic flows in real-time. One of the most famous systems belonging to the FT control type is the Traffic Network Study Tool (TRANSYT) developed by the Transport and Road Research Laboratory (TRRL) [2]. In order to obtain more details about the real-time change of traffic flows and to enhance the traffic efficiency, some advanced traffic control strategies have been developed, such as Traffic Adaptive control, where traffic flows are estimated based on the information sent by the sensors. One of the most famous Traffic Adaptive control systems is the Split Cycle Offset Optimization Technique (SCOOT) [3]. However, in all of the above control systems, each phase includes the fixed group of streams. In other words, the compatible streams cannot be grouped dynamically during the control process. Furthermore, the vehicles cannot adjust their speed profiles sufficiently in advance before arriving at the intersection, because they cannot receive the schedule of traffic signals until approaching the intersection. Then, some needless accelerations or decelerations frequently occur when the vehicles are around the intersection. Therefore, in order to excavate the potential efficiency in the intersection, many researchers try to apply Intelligent Traffic Systems (ITS) in urban transportation control.

ITS combines technologies, such as vehicle-to-infrastructure (V2I), vehicle-to-vehicle (V2V), and eco-driving to give some new strategies to improve the traffic problem. On the one hand, some studies only focus on avoiding collisions in intersections [4,5,6], with some improvement of traffic efficiency. On the other hand, more and more researchers try to get the optimal solution securely based on a certain traffic structure [7], where the control strategies are mainly based on one of the following two methods:

- In the first strategy, only the control center optimizes the vehicle passing sequence for all vehicles to decrease the stopped time before the intersection, and it is assumed that all the vehicles should stop as fast as possible before the intersection without traffic lights to wait for the right-of-way, by the following procedures: firstly, the vehicles can send their time of arrival before arriving at the intersection by the V2I; then, the control center optimizes the vehicle passing sequence for these vehicles to pass the intersection. Therefore, this strategy only optimizes the dynamic distribution of intersection resources based on the fixed arrival information of vehicles.
- In the second strategy, only the vehicles adjust their dynamic movements before the intersection to reduce the stopped time, and it is assumed that the intersection applies FT control. Through the V2I connection, vehicles can receive the scheduling signals far from the intersection. Then, the vehicles can adjust the operation before arriving at the intersection to avoid the stops. Therefore, this strategy only considers the optimization of the dynamic movement of vehicles before the intersection without cooperatively adjusting the traffic control strategies.

The first strategy is presented in the following two papers. In [8], the authors consider each vehicle separately, and apply the Branch and Bound (BB) approach to find the optimal vehicle passing sequence in the intersection, based on all the vehicles’ minimal time of arrival. In [9], the authors use Dynamic Programming (DP) to obtain the minimal final evacuation time by optimizing the vehicle passing sequence for all vehicles. For the second strategy, some examples are shown in [10,11]. The authors propose the Green Light Optimal Speed Advisory (GLOSA), where each vehicle adjusts its speed value before arriving at the intersection—according to the information sent by the intersection with FT control—to improve the possibility of encountering the green traffic light. Other similar examples for the first strategy are the eco-driving systems [12,13,14], the driver assistance system [15], and so on.

In this paper, a new cooperative traffic control algorithm is proposed by combining the above two strategies. A vehicle passing sequence is defined as an order (sequence) of allocating the right-of-way for the vehicles to cross the intersection. This sequence is presented by the travel time for vehicles to enter the intersection. The cooperation of the proposed strategy is represented by the following two-way interactions:

- On the one hand, the control center receives the range of vehicles’ times of arrival (instead of a fixed minimal time of arrival) when the vehicles arrive at the communication zone. Then, the vehicle passing sequence is optimized and sent to the vehicles by the control center.
- On the other hand, the vehicles adjust their speeds by changing the acceleration before the intersection, according to the given passing sequence, instead of the schedule in an FT control.

In the proposed cooperative optimal strategy, there are two objectives: the traffic delays and the fuel consumption. Traffic delays refers to the extra travel time for a driver or passenger to finish the trip because of the real circumstances that prevent the ideal movement of traffic [16]. It can be presented as the time difference between real travel time and the free-flow travel time, as shown in Equation (2). Fuel consumption means the total energy for the vehicle to finish the entire trip, as in Equation (23). If traffic delays are the only control objective, the maximal traffic volume is achieved and each vehicle can arrive at its destination as quickly as possible. However, sometimes the number of speed profiles for the vehicles to achieve the minimal traffic delays are not unique. The one with the minimal fuel consumption should be found. Therefore, in order to get the maximal traffic volume with the minimal fuel consumption for vehicles to finish the total travel trip, these two objectives have different priorities in the optimal operation. In other words, the traffic delays have a higher priority than the energy consumption to get the maximal traffic volume with minimal fuel consumption.

The main contributions are: (1) the proposition of a new cooperative model including a two-way interaction between the vehicles and the intersection; (2) The minimization of traffic delays with minimal fuel consumption for the vehicle to finish the whole trip; (3) The dynamic combination of the compatible streams in the optimal operation of the vehicle passing sequence; (4) The method of calculating the maximal travel speed based on the time of arrival in intersection and the calculation of minimal intersection travel time, according to the maximal travel speed for entering the intersection and the vehicle’s operation; (5) Choice of itinerary for the vehicle in the network of intersections.

Some assumptions are made as follows: (1) all vehicles are autonomous; (2) non-motorized vehicles and pedestrians are not considered in the paper; (3) delays in communication are not considered; (4) there are no traffic lights at the intersection; (5) the right-of-way is allocated to each vehicle. Each vehicle can only pass the intersection according to its right-of-way; (6) in each lane, overtaking is not allowed, which means that the rule of First In First Out (FIFO) is respected.

The paper is organized as follows: Section 1 surveys the background and literature of the traffic control; Section 2 describes the proposed dynamic model in a network of intersections; Section 3 explains the algorithm of applying DP to get the optimal vehicle passing sequence with the minimal traffic delays; Section 4 explains the optimal fuel consumption algorithm; Section 5 shows the method of choosing the itinerary for vehicles in a network of intersections; Section 6 illustrates the simulation results for the proposed algorithm and the comparison with other papers. The last section concludes the work and proposes future research.

## 2. Proposed Dynamic Traffic Modeling

**Figure 1:**
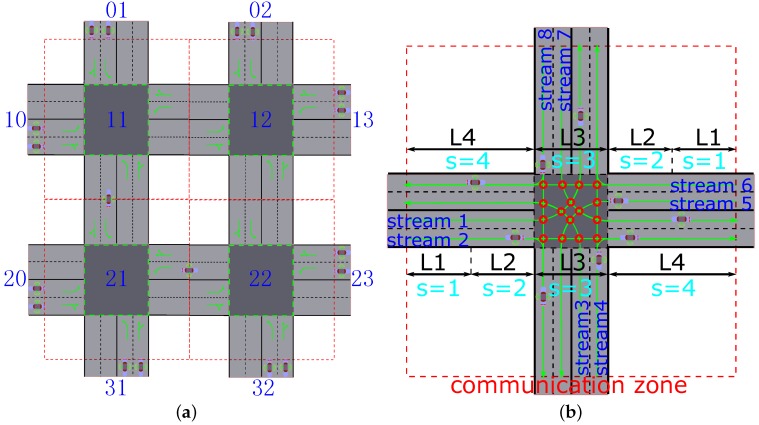
Proposed traffic model without traffic lights. (**a**) Network of intersections; (**b**) Isolated detailed intersection.

Figure 1a shows a network of intersections to be researched in the paper. In other words, this paper aims at the optimization of multiple intersections instead of an isolated intersection. However, an isolated intersection should be researched firstly, because it is the fundamental unit of the network of intersections. The communication zone in each intersection is marked by red dashed lines. Each intersection is coded according to its number of rows and columns. The intersections out of the red dashed line in the network of intersections are marked as virtual intersections (e.g., intersections 01 and 10). These virtual intersections express the origin and the destination for each vehicle. The problem to be solved in this paper is to find the optimal speed profile connecting the origin and destination with the minimal fuel consumption, without sacrificing the traffic delays for each vehicle. Therefore, the following two sub-problems should be solved:

- Method of selecting the itineraries for vehicles. That is to say, finding the list of intersections through which each vehicle passes from its origin to its destination. The method is to choose the next intersection having the lighter traffic load based on the same minimal travel distance. For example, in Figure 1a, for a vehicle from virtual intersection 20 to 02, it can choose one of the two itineraries (20-21-22-12-02 or 20-21-11-12-02) with the same minimal trip length. When this new vehicle is in intersection 21, and the total number of vehicles in intersection 22 are greater than that in 11, it chooses 11 as its next intersection, and vice versa. This operation occurs in each simulation step for the new vehicles before getting the right-of-way.
- Vehicle passing sequence in each intersection. The control center decides the vehicle passing sequence for the vehicles based on the information collected from them, with the given objectives.

In order to show the way of passing an intersection for each vehicle, Figure 1b presents a more detailed isolated intersection model having four approaches, on each of which the queue of a specific traffic stream occurs. The traffic stream refers to a part of the arrival flow of vehicles. The path employed by a traffic stream to pass through an intersection is termed as a trajectory. More than one trajectory may be used by the vehicles in some streams to traverse the intersection (e.g., there are two trajectories in Stream 4 in Figure 1b). The streams—whose trajectories do not cross—are compatible, because the vehicles from them can simultaneously traverse the intersection. Otherwise, they are incompatible streams. Figure 1b and Table 1 illustrate all the pairs of incompatible streams, whose points of crossing on the trajectory are marked as red circles; for example, the notation $l◯l^{\prime }$ means that *l* and $l^{\prime }$ are incompatible. All the notations are defined in Table 2, where the variables of suffixes, subscripts, and superscripts should be combined with other elements to present a combined notation. For example, the variable $ET3^{f}$ means the time of arrival at intersection in free-flow state, which is the lower bound of $ET3^{a}$ (real time of arrival at the intersection).

**Figure 2:**
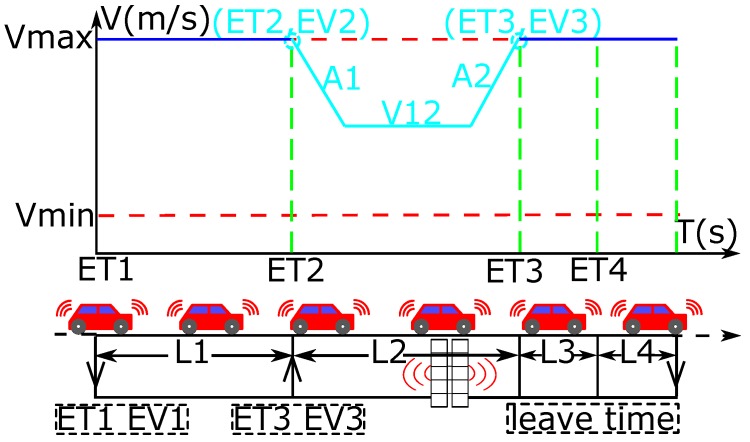
Process of traversing a communication zone.

From the view of vehicle, Figure 2 shows the processes of traversing a communication zone. When the vehicles enter the first segment, they are marked as new vehicles, and the time ET1 and the speed EV1 are sent to the control center. It is assumed that all vehicles enter the first segment with the maximal speed ($EV1=V_{max}$). Then, the vehicles always keep the maximal speed in the first segment. Once a new vehicle from any stream arrives at the second segment, it triggers an optimal process for the minimal traffic delays. An optimal process means one round of optimization of the traffic delays by applying the DP. In each optimal process, the control center only globally optimizes the new vehicles from the first segment in all the approaches to get their optimal time ET3 and speed EV3 in entering the intersection. That is to say, the control center only optimizes the new vehicles located in the first segment in each lane instead of the overall place before the intersection in order to reduce the number of vehicles requiring optimization. As a result, the calculation time can be decreased. Then, all the new vehicles should be marked as old vehicles. If the other new vehicles enter the communication zone before all the old vehicles are evacuated, these new vehicles should belong to a new optimal process without changing the vehicle passing sequence for the old vehicles, in order to avoid the sudden change of speed.

(1) $$\begin{array}{cc} \mathrm{Given}:\; & ET1,EV1 \end{array}$$

(2) $$\begin{array}{cc} \mathrm{Objective}: & \;min\left\{\sum_{l=1}^{8}\sum_{j=0}^{Nl}TD_{\left(j,l\right)}\right\};\;TD=TT^{a}-TT^{f} \end{array}$$

(3) $$\begin{array}{cc} \mathrm{Control}: & \;ET3,\;EV3 \end{array}$$

When the vehicles enter the second segment, they should adjust their speed profiles based on the optimal EV3 and ET3 sent from the control center, as shown in Figure 2. For the speed profile in this segment, the pair of variables ET3 and EV3 can form the End Point (EP), and the pair of variables ET2 and EV2 can form the Start Point (SP). Therefore, the solution is to find a speed profile connecting these two points SP and EP. In the proposed strategy, the profile is divided into three parts, because it is the minimal number to get the maximal time of arrival with the speed $VI_{max}$. Therefore, the speed profile can be represented by a series of variables V=(A1,V12,A2), where the notations A1 and A2 mean the acceleration (if it is deceleration, it should be negative). The variable V12 presents the speed in the state without acceleration. At last, when the vehicles start to pass the intersection in time ET3 with the speed EV3, they should accelerate to or keep the speed $VI_{max}$ to get the minimal intersection travel time TT3. The operation in TT4 is similar to that in the third segment.

(4) $$\begin{array}{cc} \mathrm{Given}: & \;ET1,\;EV1,\;ET3,\;EV3 \end{array}$$

(5) $$\begin{array}{cc} \mathrm{Objective}: & \;min\{FUEL\} \end{array}$$

(6) $$\begin{array}{cc} \mathrm{Control}: & \;A1,\;V12,\;A2 \end{array}$$

In general, in order to get the minimal traffic delays, the characteristics of speed profile in each segment are shown as follows: first of all, in the first segment, the vehicle should always keep the maximal speed; then, in the second segment, the vehicle should adjust its operation to meet the given time ET3 and speed EV3 for entering the intersection; next, in the third segment, the vehicle should accelerate to or keep the $VI_{max}$; finally, in the fourth segment, the vehicle should accelerate to or keep the $V_{max}$. Therefore, the vehicles can only optimize the fuel consumption in the second segment, because the control strategies of vehicles in the other segments are fixed (acceleration to or keeping the maximal allowed speed).

## 3. Minimization of Time Delays by Applying Dynamic Programming

This section presents the model of achieving the minimal traffic delays for the vehicles by optimizing their vehicle passing sequence in the intersection, based on the security restrictions presented in Section 3.3. Therefore, the proposed strategy considering the dynamic movement of each vehicle has to solve the following sub-problems:

- Calculation of the range of ET3, which means the reasonable time period for the vehicle to arrive at the intersection. In this time range, the key point is the lower bound, which is the minimal time for the vehicle to arrive at the intersection. This lower bound is the time $ET3^{f}$, which is achieved by the vehicle to travel in the free-flow state, because it can always travel with the maximal allowed speed in this state. Thus, it is impossible for the vehicles to arrive at the intersection before the time $ET3^{f}$.
- Calculation of the higher EV3 based on the ET3. The variable ET3 presents the time when the vehicle is allowed to pass the intersection. However, for a given ET3, there are countless possibilities of EV3 with which the vehicle can start to pass the intersection. Therefore, in the proposed strategy, the ET3 with the higher value is chosen to reduce the intersection travel time TT3. The higher EV3 is, the smaller the time needed by vehicles to accelerate to $VI_{max}$. As a result, the minimal value of TT3 can be achieved. Referring to sub-Section 3.1.
- Calculation of the minimal TT3 according to the maximal EV3 and the vehicles’ operations in the intersection. The reason that the variable of maximal EV3 affects the minimal TT3 is explained in the above step. The length for the vehicle to pass the intersection is various in different operations. For example, the length for the vehicle to turn left in the intersection is longer than that in the operation of turning right. The vehicle’s operation in the intersection depends on its origin, its destination, and the traffic volume in the adjacent intersections, as shown in Section 5. Referring to Section 3.2.
- The mathematical expressions of the security restrictions during the optimal process for the vehicle passing sequence. Referring to Section 3.3.
- The process of optimizing the vehicle passing sequence by DP. Referring to Section 3.4.

These sub-problems are described in detail in the following sub-sections.

### 3.1. Calculation of the Maximal EV3 Based on the ET3

The methods for calculating the maximal EV3 depend on the period where the time ET3 locates. Then, the reasonable range of ET3 is divided into four parts based on the Threshold Limit Value (TLV) of EV3 $\left(TLV=\left\{VI_{max},\;V_{min},\;0\right\}\right)$. These three different values of TLV correspond to three key points of ET3 (KP={KP1,KP2,KP3}). The length in the second segment L2 should be long enough for the vehicle to decelerate from $V_{max}$ to $V_{min}$, and to accelerate from $V_{min}$ to $V_{max}$ ($V_{max}\geq VI_{max}$). This rule is for the reason of readability, because the method of calculating the speed profile is similar when the L2 is not long enough.

(7) $$\begin{array}{cc} L_{2}\geq & \left(V_{min}^{2}-V_{max}^{2}\right)/\left(2D_{max}\right)+\left(V_{max}^{2}-V_{min}^{2}\right)/\left(2A_{max}\right) \end{array}$$

Next, the equations for calculating the variables KP1,KP2, and KP3 are presented.

#### 3.1.1. KP1—TLV ($V_{max}$)

KP1 expresses the maximal time at which the vehicle can arrive at the intersection with $VI_{max}$. In other words, it is impossible for the vehicle to arrive at the intersection with $VI_{max}$ after time KP1. The vehicle control strategies for obtaining KP1 are as follows: first, the vehicle decelerates to $V_{min}$ with $D_{max}$; second, it maintains $V_{min}$; finally, it accelerates to $VI_{max}$ with $A_{max}$ in time KP1.

(8) $$\begin{array}{cc} KP1= & ET2+\left(V_{min}-V_{max}\right)/D_{max}+\left(VI_{max}-V_{min}\right)/A_{max}+\\ & \left(L2-\left(V_{min}^{2}-V_{max}^{2}\right)/\left(2D_{max}\right)-\left(VI_{max}^{2}-V_{min}^{2}\right)/\left(2A_{max}\right)\right)/V_{min} \end{array}$$

#### 3.1.2. KP2—TLV ($V_{min}$)

KP2 means the maximal time of arriving at intersection with $V_{min}$ for a vehicle. That is to say, after time KP2, it is impossible for the vehicle to arrive at the intersection with a speed that is greater than $V_{min}$. The vehicles apply the following operations to achieve the value of KP2: first of all, the vehicle decelerates to $V_{min}$ with $D_{max}$, then it keeps this speed until arriving at the intersection.

(9) $$\begin{array}{c} KP2=ET2+\left(V_{min}-V_{max}\right)/D_{max}+\left(L2-\left(V_{min}^{2}-V_{max}^{2}\right)/\left(2D_{max}\right)\right)/V_{min} \end{array}$$

#### 3.1.3. KP3—TLV (0)

KP3 presents the time after which the vehicle must stop before the intersection. The following control strategies are used by vehicles to obtain KP3: first of all, the vehicle decelerates to $V_{min}$ with $D_{max}$, then it keeps $V_{min}$, finally, it decelerates to stop before the intersection with $D_{max}$.

(10) $$\begin{array}{c} KP3=ET2-V_{max}/D_{max}+\left(L2+V_{max}^{2}/\left(2D_{max}\right)\right)/V_{min} \end{array}$$

**Figure 3:**
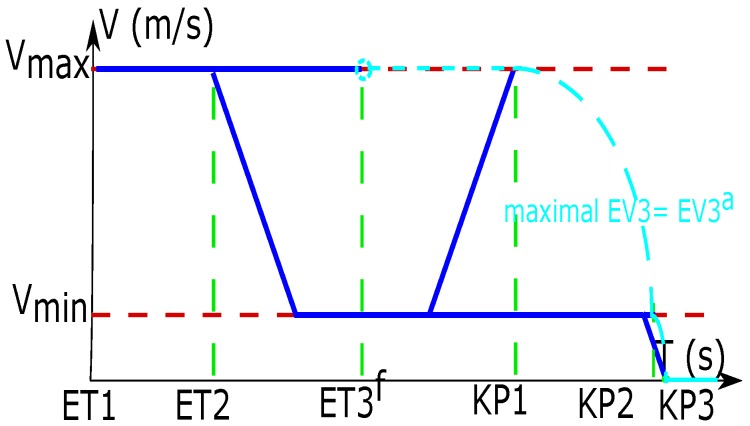
Calculation of the maximal EV3 based on the $ET3^{a}$.

After the above three key points have been calculated, the maximal EV3 in each segment can be calculated based on the real value of ET3 ($ET3^{a}$). The maximal EV3 can be also represented by $EV3^{a}$, because it is chosen by the vehicle as the real travel speed to enter the intersection. The profile between the $EV3^{a}$ and ET3 is shown in Figure 3 and summarized in Table 3.

(11) $$\begin{array}{c} EV3^{a}=V_{min}+\sqrt{A_{max}\left({\left(V_{max}-V_{min}\right)}^{2}/D_{max}+2\left(L2-V_{min}TT2\right)\right)} \end{array}$$

(12) $$\begin{array}{c} EV3^{a}=V_{min}-\sqrt{{\left(V_{max}-V_{min}\right)}^{2}+2D_{max}\left(L2-V_{min}TT2\right)} \end{array}$$

### 3.2. Calculation of the Minimal TT3

**Figure 4:**
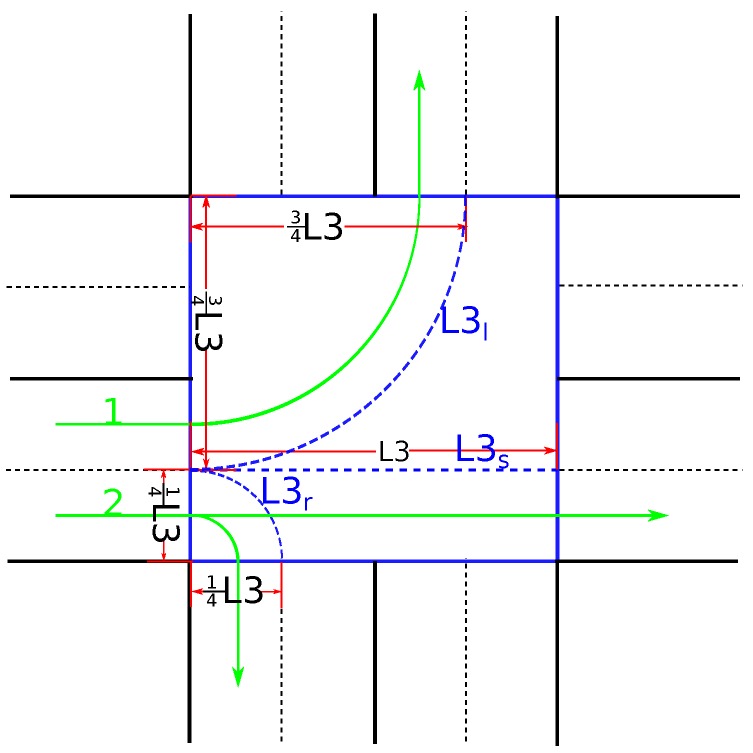
Various lengths of passing the intersection in different operations.

There are two factors affecting the minimal TT3. The first factor is the length for the vehicle to pass the intersection $L3_{o}$ (operation(o)={r,s,l}), which depends on the vehicle’s operation; i.e., going straight (s), turning right (r), or turning left (l) (as shown in Figure 4). The method of calculating this length is expressed in Equations (13)–(15). The method for calculating $L3_{o}$ in the other streams is similar to that in streams 1 and 2.

(13) $$\begin{array}{cc} L3_{r} & =\pi L3/8 \end{array}$$

(14) $$\begin{array}{cc} L3_{s} & =L3 \end{array}$$

(15) $$\begin{array}{cc} L3_{l} & =3\pi L3/8 \end{array}$$

The second factor is $EV3^{a}$, which affects the running speed in the intersection. First of all, the Minimal Distance (MD) should be calculated, which refers to the length needed by the vehicle to accelerate from $EV3^{a}$ to $VI_{max}$ with $A_{max}$. If $L3_{o}$ is longer than MD, there is enough travel distance for the vehicle to accelerate to $VI_{max}$ in the intersection. After arriving at the speed $VI_{max}$, this vehicle keeps the state to finish the remainder of the travel distance in the intersection. Otherwise, it keeps the acceleration in the whole travel distance of the $L3_{o}$. As a result, the formulations for calculating the variables $TT3^{a}$, $EV4^{a}$, and $TT4^{a}$ are shown in Equations (17)–(19), respectively.

(16) $$\begin{array}{cc} MD & =\left(VI_{max}^{2}-{\left(EV3^{a}\right)}^{2}\right)/\left(2A_{max}\right) \end{array}$$

(17) $$\begin{array}{cc} TT3^{a} & =\left\{\begin{array}{cc} \left(VI_{max}-EV3^{a}\right)/A_{max}+\left(L3_{o}-\left(VI_{max}^{2}-{\left(EV3^{a}\right)}^{2}\right)/\left(2A_{max}\right)\right)/VI_{max} & \;\mathrm{if}\;L3_{o}>MD\\ \left(-EV3^{a}+\sqrt{{\left(EV3^{a}\right)}^{2}+2A_{max}L3_{o}}\right)/A_{max} & \;\mathrm{if}\;L3_{o}\leq MD \end{array}\right. \end{array}$$

(18) $$\begin{array}{cc} EV4^{a} & =\left\{\begin{array}{cc} VI_{max} & \;\mathrm{if}\;L3_{o}>MD\\ EV3^{a}+TT3^{a}*A_{max} & \;\mathrm{if}\;L3_{o}\leq MD \end{array}\right. \end{array}$$

(19) $$\begin{array}{cc} TT4^{a} & =\left(V_{max}-VI_{max}\right)/A_{max}+\left(L4-\left(V_{max}^{2}-VI_{max}^{2}\right)/\left(2A_{max}\right)\right)/V_{max} \end{array}$$

### 3.3. The Mathematical Expressions of the Security Restrictions

**Figure 5:**
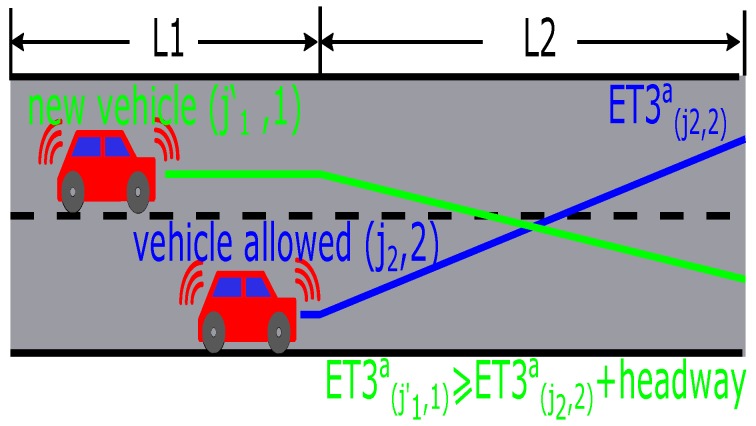
Lane change model.

In the traditional method, the security restrictions are satisfied by fixedly grouping the streams as a phase and applying a fixed phase sequence, which prevents the further improvement of efficiency in the intersection. However, in this paper, the combinations and sequences of compatible streams are dynamic. Therefore, for each vehicle, the calculation of time $ET3^{a}$ should consider the real-time situation in all streams, which can be classified as the following three groups:

- Incompatible streams. A new vehicle is permitted to start to pass the intersection iff the other vehicles located before it in the vehicle passing sequence and coming from incompatible streams have passed the intersection completely ($ET3_{\left(j,l\right)}^{a}\geq ET3_{\left(j^{\prime },l^{\prime }\right)}^{a}+TT3_{\left(j^{\prime },l^{\prime }\right)}^{a}\left(l^{\prime }◯l\right)$, vehicle $l_{j}$ is behind the other one $l_{j^{\prime }}^{\prime }$ in the vehicle passing sequence). This rule can exploit the intersections’ resources more effectively and dynamically.
- The same lane. The minimal headway should be respected ($|ET3_{\left(j,l\right)}^{a}-ET3_{\left(j^{\prime },l^{\prime }\right)}^{a}|\geq HW\left(l^{\prime }=l\right)$).
- The other lane in the same streams. For one vehicle, if its original lane does not correspond to its operation in the intersection, it has to change lanes in the second segment. The minimal headway should be considered when two vehicles cross, as shown in Figure 5.

### 3.4. Process of Applying Dynamic Programming to Optimize the Vehicle Passing Sequence

The DP is applied to get the optimal vehicle passing sequence with the objective of the minimal traffic delays, based on the security restrictions. The problem of finding this sequence is decomposed to many sub-problems, in each of which only one vehicle is treated. Then, the traffic delays of the current sub-problem equal to the sum of the traffic delays of the last vehicle treated and the traffic delays of the previous sub-problem. Therefore, the traffic delays of the last vehicle treated depend on its previous sub-problem, among which the one with the minimal traffic delays is selected. The formula of DP recursion is given as follows:

(20) $$\begin{array}{c} TD\left(j_{1},...,j_{8},l\right)=\min_{l^{\prime }\in \left\{1,8\right\}}\left\{TD\left(j_{1}^{\prime },...,j_{8}^{\prime },l^{\prime }\right)+TD_{\left(j_{l},l\right)}\right\} \end{array}$$

where $j_{1}^{\prime }=j_{1}$ for $l^{\prime }\neq l$; otherwise, $j_{1}^{\prime }=j_{1}-1$ for $l^{\prime }=l$, l∈[1,8]. The recursion continues until reaching the initialization conditions (only one vehicle that is not included in the sequence in all lanes). That is to say, the recursion stops when there is only one vehicle untreated in all lanes.

(21) $$\begin{array}{cc} TD(j_{1},...,j_{8},l)= & \left\{\begin{array}{c} TD_{\left(j_{l},l\right)}:\sum_{l^{\prime }=1}^{8}j_{l^{\prime }}=1\&j_{l}=1\\ \infty :\sum_{l^{\prime }=1}^{8}j_{l^{\prime }}=1\&j_{l}=0 \end{array}\right. \end{array}$$

**Figure 6:**
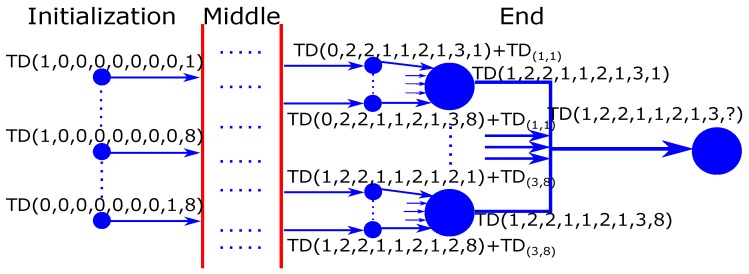
Example for the process of Dynamic Programming (DP) recursion.

Equation (21) is explained by two examples. The first one is the initial sub-problem of TD(1,0,0,0,0,0,0,0,1), which means that only the first vehicle in lane 1 is included in the sub-problem, and the vehicle treated in the current iteration is located in lane 1. This situation accords with reality, and the total traffic delays are equal to the traffic delays of the first vehicle in lane 1. However, the second situation is impossible, TD(1,0,0,0,0,0,0,0,2), which means that the number of vehicles in lane 2 that are included in the sequence is zero. However, the vehicle treated in the current iteration is located in lane 2. If it is contradictory to the reality, then the total traffic delays are considered as infinite (∞). Figure 6 illustrates Equations (20) and (21), with the example of traffic lanes containing 1, 2, 2, 1, 1, 2, 1, 3 vehicles, respectively.

## 4. Minimization of Fuel Consumption by Adjusting the Speed Profile in the Second Segment

This section presents the method of getting the speed profile with minimal fuel consumption for each vehicle, based on the pair of variables (ET3, EV3) obtained in Section 3.

The speed profile in the second segment should be optimized iff $EV3=VI_{max}$, because there is only one possible speed profile if EV3 is smaller than $VI_{max}$.

Containing a model of fuel consumption is indispensable for optimizing fuel consumption [12]. There are many fuel consumption models in the literature, which include the Virginia Tech (VT) comprehensive power-based fuel model (VT-CPFM) [17], the Virginia Tech microscopic (VT-Micro) model [18], and the vehicle drive line model [19]. In this paper, the VT-Micro model is chosen, because the VT-Micro model, which is expressed in Equation (22), can calculate the fuel consumption by applying the instantaneous speed and acceleration, instead of the average value.

(22) $$\begin{array}{cc} MOE(v,a) & =\left\{\begin{array}{c} e^{\sum_{x=0}^{3}\sum_{y=0}^{3}\left(L_{\left(x,y\right)}\times v^{x}\times a^{y}\right)}:a\geq 0\\ e^{\sum_{x=0}^{3}\sum_{y=0}^{3}\left(M_{\left(x,y\right)}\times v^{x}\times a^{y}\right)}:a<0 \end{array}\right. \end{array}$$

MOE(v,a) is the instantaneous fuel consumption at the speed power *v* and acceleration power *a*. $L_{x,y}$ and $M_{x,y}$ are VT-Micro regression coefficients. Therefore, VT-Micro is a discrete model for the calculation of fuel consumption, and the continuous speed profile should be discretized to calculate its fuel consumption. Each part of time period $t_{\left(p,h\right)}$ is divided into $\left\lceil N_{p}\right\rceil$ units by time step $t_{step}$. Then, the fuel consumption functions for the vehicle in the second segment are achieved in Equations (23)–(26). For the other segments, the function calculating the fuel consumption is similar to that in the second segment.

(23) $$\begin{array}{cc} FUEL & =\sum_{p=1}^{3}\sum_{h=1}^{N_{p}}MOE\left(v_{\left(p,h\right)},a_{p}\right)*t_{\left(p,h\right)} \end{array}$$

(24) $$\begin{array}{cc} v_{\left(p,h\right)} & =v_{\left(p,h-1\right)}+a_{p}*t_{\left(p,h\right)} \end{array}$$

(25) $$\begin{array}{cc} t_{\left(p,h\right)} & =min\;(t_{step},\;tp-t_{step}*\left(h-1\right)) \end{array}$$

(26) $$\begin{array}{cc} N_{p} & =\lceil tp/t_{step}\rceil \end{array}$$

where $v_{\left(1,0\right)}=V_{max}$, $v_{\left(2,0\right)}=v_{\left(3,0\right)}=V12$.

Among the countless speed profiles satisfying the ET3 and EV3, the one with the minimal fuel consumption is chosen by an exhaustive search with V=(A1,V12,A2) $(A1\in \left[D_{max},0\right]$, $V12\in [V_{min},\;V_{max}]$, $A2\in \left[0,A_{max}\right]\left(V12<VI_{max}\right)\;or\;A2\in \left[D_{max},0\right]\left(V12\geq VI_{max}\right))$.

## 5. Choice of Itinerary for Each Vehicle in the Network of Intersections

The section presents the algorithm for choosing a list of intersections dynamically as an itinerary for vehicles to arrive at their destinations from their origins. In other words, each vehicle must choose some intersections to connect its origin to the destination. As shown in Figure 1a, for the reason of simplicity, there are only two intersections in each row and each column in the network of intersections, respectively. The traffic control strategy introduced in the paper can be applied to a larger network.

**Figure 7:**
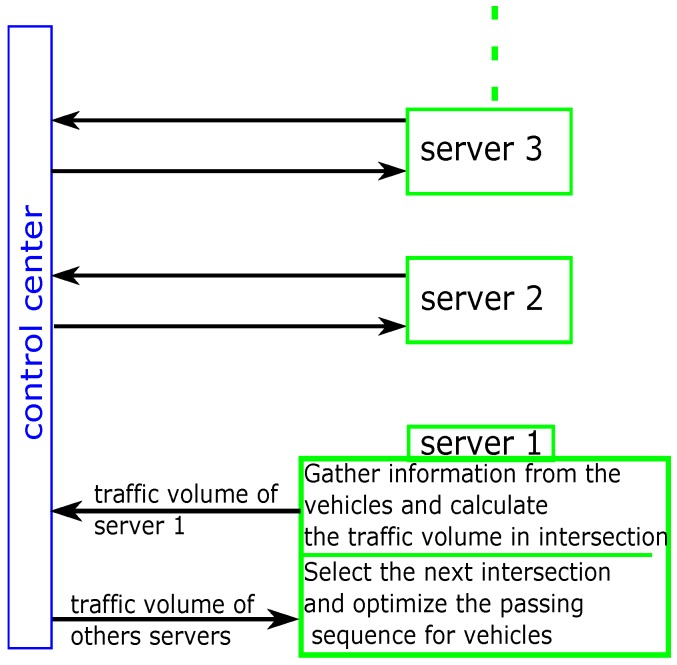
The exchange of information between the network and intersections.

In this network, there is a small control server in each intersection and a large control center in the network to coordinate the small servers. The small servers gather the information from approaching vehicles and calculate the real-time traffic loads (total number of vehicles in current intersection) in the intersections. Then, the control center receives this information and distributes it to each small server in each simulation step. In other words, the traffic loads in each intersection should be updated in each simulation step. As a result, each small server can get the real-time traffic loads of the other intersections. Finally, each small server chooses the next intersection for its vehicles based on traffic loads in the other adjacent intersections and the destination of its vehicles. Figure 7 shows the above process.

When we try to find an itinerary for vehicles to finish the trip, we do not choose all of the intersections for the vehicle to pass through at one simulation step, because the traffic loads in each intersection change rapidly during the simulation process. For example, at first, some intersections are vacant. Then, many vehicles prefer to finish their trip by passing through these intersections, which may lead to traffic congestion in these intersections. Therefore, for each vehicle, we only give the advice of choosing the next intersection based on its destination, current intersection, and the traffic loads in the adjacent intersections. The principles for electing the best itinerary are as follows:

- The travel distance is put in the first place. In other words, all vehicles try to find an itinerary with the minimal travel distance.
- The traffic loads in the adjacent intersections are the second element. The traffic loads in the virtual intersection is defined as infinite. If the itinerary with the same travel distance is not unique, the vehicle chooses the one where the next intersection has the least traffic loads in order to reduce the traffic delays in trip.

As a result, the vehicle can adjust its itinerary dynamically in each intersection based on the real traffic circumstances to decrease the traffic delays without augmenting the travel distance. Then, the operation for the vehicle in the current intersection is based on its next optimal intersection in the itinerary. For example, we take the vehicles which enter the intersection from the east to explain the above method. In Algorithm 1, lines 1–8 mean the cases where there is only one itinerary with the minimal travel distance, and the vehicles have to choose this itinerary. Lines 9–20 present the method of choosing the next intersection with the smaller traffic loads, when there is more than one itinerary having the minimal travel distance.

The overall process of the proposed traffic control model is shown in Algorithm 2. In lines 4–7, each intersection communicates with the others in the network to exchange the vehicles and the traffic loads. In lines 8–14, when a new optimal process is triggered, each intersection optimizes the vehicle passing sequence for its new vehicles, and each new vehicle adjusts its speed profile in the second segment.

**Algorithm 1:** The algorithm for choosing the itinerary for each vehicle to enter the intersections from the east.
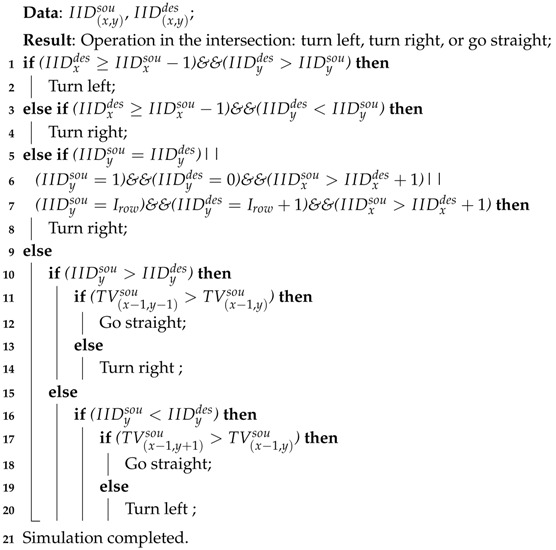


**Algorithm 2:** The overall process of the proposed algorithm.
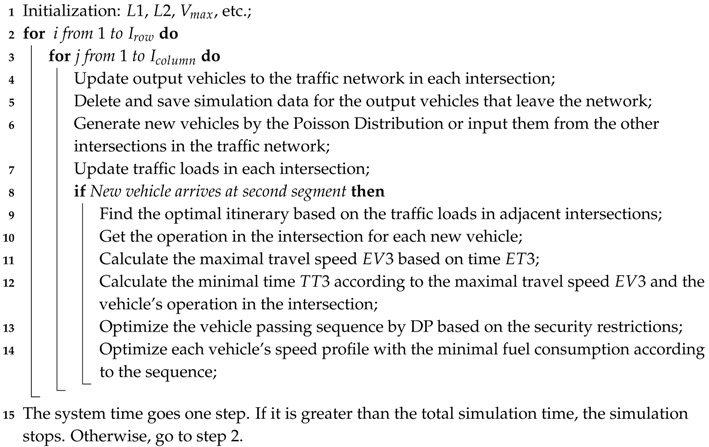


## 6. Results

A series of computational simulations are performed. The results are compared with papers [9,10] to evaluate the performance of the proposed algorithm. The simulation system is coded by C++ and run on a desktop computer with eight 3.4 GHz Intel processors. In each virtual intersection, the generation of the new vehicles is assumed to obey the Poisson Distribution, which accurately represents the actual traffic system [20,21,22]. For each new vehicle generated in a virtual intersection (e.g., 01), its possible destinations (other virtual intersections, such as 02, 10, 20, etc.) are the same. The initial speed for each vehicle is the maximal speed of the road ($V_{max}$). The other parameters are shown in Table 4. The units for each type of variable are: time (s), speed (m/s), traffic volume in each stream (veh/h), and length (m). The simulation results are compared with other papers in different traffic volumes by several criteria: stopped time before the intersection, intersection travel time, and traffic delays (s/veh/intersection); travel speed for entering the intersection (m/s/veh/intersection); fuel consumption (mL/m), calculation time for the optimization of traffic delays and fuel consumption (s/time).

### 6.1. Comparison with the Work Proposed by Abbas-Turki et al.

In the paper [9], the authors (Abbas-Turki et al.) diminish the stopped time by optimizing the vehicle passing sequence in the intersection, like CTCVI. However, the paper [9] assumes that all the vehicles should stop before the intersection, and the time of arrival is fixed. The range in each optimization is the whole communication zone before the intersection; as a result, the optimal space is too large to solve when the intersection dimension and traffic volume are large. Therefore, in this comparison with the paper [9], the traffic volume is only 100 veh/h in an isolated intersection.

From Table 5, the proposed strategy is better than paper [9] in all major criteria, for the following reasons:

- The control center optimizes the vehicle passing sequence based on the range of time of arrival for each vehicle, to assure the validity of the solution obtained, instead of the fixed time of arrival. Additionally, each vehicle optimizes its speed profile according to the permission given by the control center.
- Owing to the first reason, each vehicle can enter the intersection with a higher speed, avoid stopping before it, and take less time to pass it, meaning that it can evacuate the vehicles more rapidly.
- The proposed strategy avoids needless decelerations before the intersection to save fuel consumption and travel time.
- The proposed strategy can decrease the complexity of optimization by considering a smaller part of range optimal without reducing the performance, which is proven by the calculation time for the traffic delays.

### 6.2. Comparison with the Paper Proposed by K. Katsaros et al.

In the paper [10], the authors (K. Katsaros et al.) propose the Green Light Optimal Speed Advisory (GLOSA) algorithm, where each vehicle optimizes the speed profile based on the schedule of signals sent from the intersection with a FT control by the V2I connection. As a result, the probability for vehicles to encounter the green signals in the intersection can be increased to reduce the stopped time and fuel consumption. However, Reference [10] does not optimize the traffic control cooperatively.

**Figure 8:**
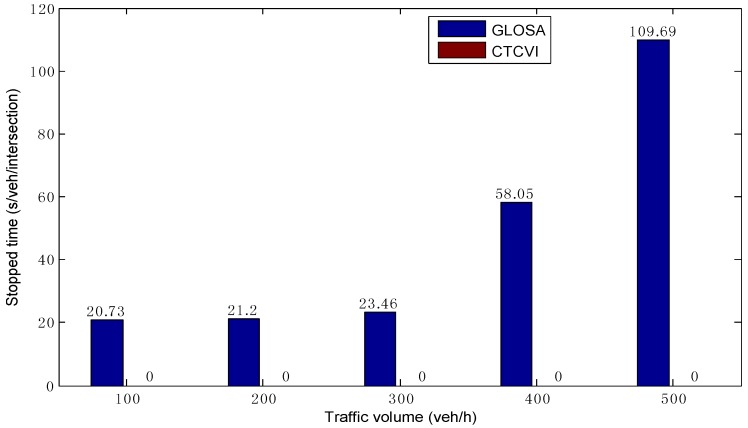
Comparison of average stopped time before the intersection.

As shown in Figure 8, the stopped time in the CTCVI strategy is always zero in different traffic volumes, which is smaller than in GLOSA. This means that all vehicles can avoid stopping before crossing the intersection in the CTCVI, because the CTCVI dynamically groups the compatible streams based on the different vehicles’ time of arrival, and allocates the right-of-way precisely to each vehicle instead of setting the fixed phase, green time, and phase sequence. Therefore, the CTCVI has a higher effectiveness in helping the vehicles reduce the stopped time before the intersection compared to GLOSA.

**Figure 9:**
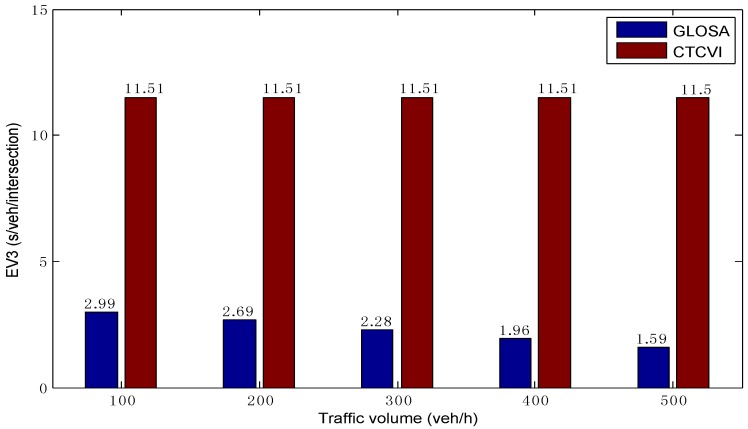
Average travel speed for entering the intersection.

**Figure 10:**
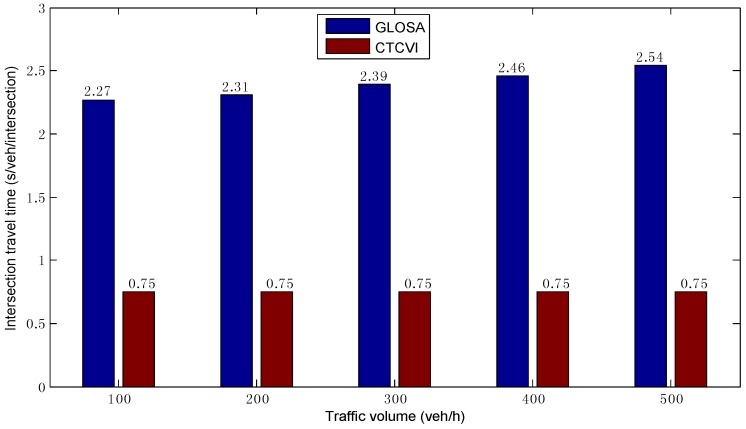
Comparison of average intersection travel time.

As shown in Figure 9, in different traffic volumes, the average EV3 in the CTCVI is almost the same, because all vehicles can enter the intersection with the speed $VI_{max}$. Additionally, it is higher than the EV3 in the GLOSA, because the CTCVI always tries to find the speed $VI_{max}$ for each vehicle based on the actual ET3, rather than just finding one possible speed, like the GLOSA. This is a key point in reducing the intersection travel time TT3, because each vehicle can pass the intersection more quickly with a higher travel speed, as Figure 10 shows. The average TT3 is almost the same in the CTCVI, due to the fact that each vehicle can keep the $VI_{max}$ in crossing the intersection. Additionally, it is smaller than the average TT3 in GLOSA, owing to the higher EV3 in the CTCVI. As a result, the intersection can be shared more efficiently by the traffic streams in the CTCVI.

**Figure 11:**
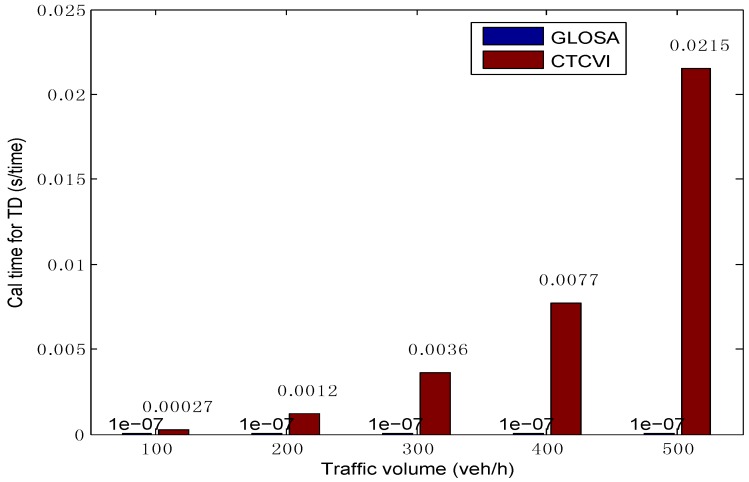
Comparison of calculation time in the optimization of traffic delays.

**Figure 12:**
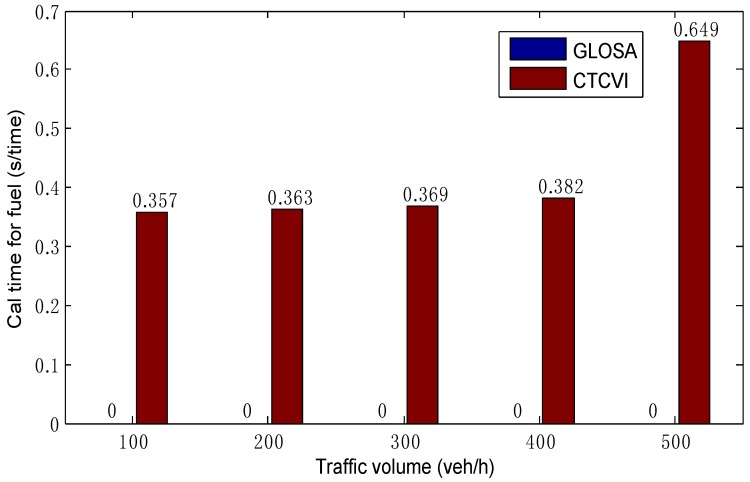
Comparison of calculation time in the optimization of fuel consumption.

The average time of computational simulation includes two parts. The first part is the calculation time for optimizing the traffic delays. Although it increases rapidly with the augment of traffic volume, its value is small and could satisfy the need of real-time calculation, as Figure 11 shows. This means that, in the optimization of traffic delays, the proposed model satisfies the real-time demand, and can reduce the complexity optimization without decreasing the control performance, thanks to a more cooperative control. The second part is the calculation time for the optimization of fuel consumption, as shown in Figure 12. It takes some time for the CTCVI to calculate the fuel consumption in the second segment, because the CTCVI executes an exhaustive search to find the solution, which can be improved by applying a heuristic method (such as genetic algorithm) to get an approximate optimal solution.

**Figure 13:**
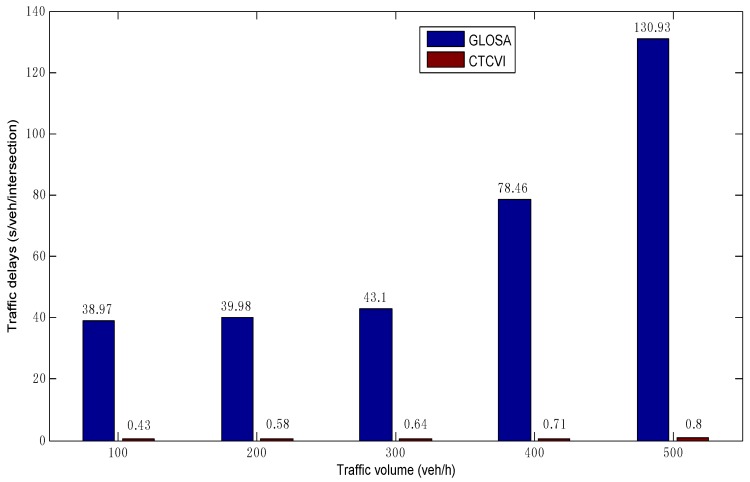
Comparison of average traffic delays.

Figure 13 shows the comparison of average traffic delays. The traffic delays are defined as the time difference between actual travel time and free-flow travel time for each vehicle, as expressed in Equation (2). The average traffic delays in the CTCVI are smaller than 1 s in different traffic volumes, which proves that most vehicles can almost travel in free-flow state. With the increase of the traffic volume, the traffic delays in the CTCVI raise more slowly than that in GLOSA, since the vehicles can enter the intersection with a higher speed and expend less intersection travel time in the CTCVI, as shown in Figure 9 and Figure 10. The CTCVI can save traffic delays by at least 98.9% in the traffic volume 100 veh/h and at most by 99.4% in the traffic volume 500 veh/h, compared with that in the GLOSA.

**Figure 14:**
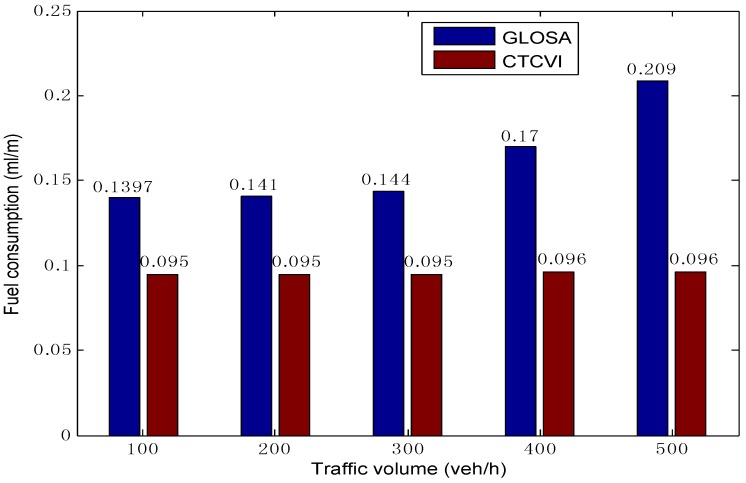
Comparison of average fuel consumption.

The CTCVI can have a better performance in the optimization of fuel consumption (as Figure 14 shows) compared to GLOSA, for the following reasons: (1) the vehicles have a smaller stopped time, as Figure 8 shows. As a result, they can avoid the stop-and-go pattern that leads to excessive fuel consumption; (2) The vehicles look for the speed profile with the minimal fuel consumption in the second segment, instead of only finding a reasonable solution. With the increase of the traffic volume, the CTCVI can save fuel consumption from 28.78% to 49.28%, according to the comparison with the GLOSA.

In brief, with the increase of traffic flow, the CTCVI can always achieve a good performance. Therefore, the CTCVI model is better than the GLOSA model in the major criteria in different traffic volumes, which depends on the reasons as follows:

- the intersection and the vehicles are collaboratively controlled. This is two-way cooperation, instead of one-way one. As a result, the vehicles change their speeds dynamically according to the traffic control strategy to reduce the traffic delays.
- Each vehicle always finds the maximal travel speed to reduce the intersection travel time as much as possible.

## 7. Conclusions

In this paper, a cooperative control algorithm in a network of intersections without traffic signals is proposed. This algorithm can achieve the minimal fuel consumption based on the minimal traffic delays for all vehicles. It properly exploits the advantage of V2I to cooperatively control the vehicles and the intersections by the following procedures. First of all, all of the vehicles entering the communication zone calculate the profiles of their maximal EV3 based on the ET3, and the minimal TT3 according to the maximal EV3. The above two calculations point out that the proposed model considers each vehicle independently, and precisely take into account its movement in order to calculate the security in the intersection and the total traffic delays. Then, the information is sent to the control center, based on which, the vehicle passing sequence is optimized by the DP. Finally, each vehicle optimizes its speed profiles according to this sequence in the second segment.

The simulation results show that the proposed strategy is more effective than References [9,10] in both traffic delays and fuel consumption. Compared with paper [9], the optimal range in the proposed strategy is a part of the communication zone instead of all places before the intersection, enormously reducing the complexity, and the proposed strategy makes a vehicle optimize the speed profile before the intersection to avoid unnecessary deceleration. Compared with paper [10], the proposed strategy executes a two-way cooperation to improve the traffic performance, and the control center finds the optimal vehicle passing sequence by dynamically combining the compatible streams under the security restrictions.

In the future, the method of choosing the itinerary should be improved in a grander network of intersections, because optimizing the itinerary for all vehicles can avoid congestion in the intersections, and take advantage of the network resources more effectively. The priority for some urgent vehicles should also be considered, such as police cars, ambulances, and so on.

## Figures and Tables

**Table 1:** All the pairs of incompatible streams.

Streams	1	2	3	4	5	6	7	8
1			◯			◯	◯	◯
2			◯	◯	◯			◯
3	◯	◯			◯			◯
4		◯			◯	◯	◯	
5		◯	◯	◯			◯	
6	◯			◯			◯	◯
7	◯			◯	◯	◯		
8	◯	◯	◯			◯		

**Table 2:** Definitions of the notations.

Notations	Definitions
*l*	The index of lane in approaches, l∈[1,8]
Nl	The number of new vehicles on lane *l*.
$\left(j_{l},l\right)$	***Subscripts***. The $j_{l}$-th vehicle on lane *l*, $j_{l}\in \left[0,Nl\right]$.
$\left(j_{1},...,j_{8},l\right)$	***Subscripts***. The $j_{l^{\prime }}$-th vehicle on the lane $l^{\prime }$ is included in the vehicle passing sequence, respectively, $l^{\prime }\in \left[1,8\right]$. The last one comes from the lane *l*.
*a*, *f*	***Superscripts***. The value in the actual flow state or the free-flow state, respectively.
*l*, *r* and *s*	***Superscripts***. These refer to the following operations in the intersection: turn left, turn right, and go straight, respectively.
*s*	The *s*-th section of the communication zone, s∈[1,4].
ETs	The time of arrival at the *s*-th section, s∈[1,4].
EVs	The travel speed entering the *s*-th section.
TTs, TT	The travel time in *s*-th section and in all sections: TT=TT1+...+TT4.
TD, FUEL	The traffic delays or fuel consumption for the entire trip.
HW	Headways, which refer to the time (in seconds) between two successive vehicles when they get through the same point on the road.
Ls	The length of *s*-th section.
$A_{max},D_{max}$	The maximal acceleration and deceleration for each vehicle.
$V_{max},V_{min}$	The speed limit on the road (except for the intersection): maximum and minimum.
$VI_{max}$	The speed limit (maximum) on the intersection.
$I_{row}$, $I_{column}$	The dimension of the intersection network: number of rows and columns, respectively.
$t_{step}$	The time step in the simulation.
$IID_{\left(x,y\right)}^{sou}$, $IID_{\left(x,y\right)}^{des}$	The coordinate of intersection presenting the origin or the destination for each vehicle, referring to Figure 1a.

**Table 3:** Calculation of the maximal EV3 based on the $ET3^{a}$.

Interval of $\mathrm{ET}3^{a}$	Range of $\mathrm{EV}3^{a}$	Formulation of Calculating $\mathrm{EV}3^{a}$
$\left[ET3^{f},KP1\right]$	$VI_{max}$	$VI_{max}$
(KP1,KP2]	$\left[V_{min},VI_{max}\right)$	Equation (11)
(KP2,KP3)	$\left(0,V_{min}\right]$	Equation (12)
[KP3,∞)	0	0

**Table 4:** Simulation parameters.

$V_{\max}$	$V_{\min}$	${\mathrm{VI}}_{\max}^{l}$	${\mathrm{VI}}_{\max}^{r}$	${\mathrm{VI}}_{\max}^{s}$	TV	$I_{\mathrm{cow}}$	$I_{\mathrm{column}}$
14	4	0.8 $V_{max}$	0.6 $V_{max}$	$V_{max}$	500	2	2
$L_{1}$	$L_{2}$	$L_{3}$	$L_{4}$	HW	$t_{step}$	$A_{max}$	$D_{max}$
100	200	10	300	1	0.1	2	-2

**Table 5:** Comparison between the CTCVI and Reference [9].

**Traffic Delays**		**Fuel Consumption**		**Travel Speed for Entering Intersection**	
Paper [9]	CTCVI	Paper [9]	CTCVI	Paper [9]	CTCVI
6.42	0.43	0.129	0.095	0	11.51
**Intersection Travel Time**		**Stopped Time**		**Calculation Time for the Optimization of** TD	
Paper [9]	CTCVI	Paper [9]	CTCVI	Paper [9]	CTCVI
2.88	0.75	5.25	0	0.0087	0.00029

## Abbreviations

The following abbreviations are used in this manuscript:

DP	Dynamic Programming
GLOSA	Green Light Optimal Speed Advisory
CTCVI	Cooperative Traffic Control of Vehicle-Intersection
